# Complete Genome Sequence of a *bla*_OXA-58_-Producing *Acinetobacter baumannii* Strain Isolated from a Mexican Hospital

**DOI:** 10.1128/genomeA.00949-17

**Published:** 2017-09-07

**Authors:** Ángeles Pérez-Oseguera, Semiramis Castro-Jaimes, Abraham David Salgado-Camargo, Jesus Silva-Sanchez, Elvira Garza-González, Santiago Castillo-Ramírez, Miguel Ángel Cevallos

**Affiliations:** a Programa de Genómica Evolutiva, Centro de Ciencias Genómicas, Universidad Nacional Autónoma de México, Cuernavaca, Morelos, Mexico; b Grupo de Resistencia Bacteriana, Centro de Investigaciones Sobre Enfermedades Infecciosas, Instituto Nacional de Salud Pública, Cuernavaca, Mexico; c Servicio de Gastroenterología, Hospital Universitario Dr. José Eleuterio González, Nuevo León, Mexico

## Abstract

In this study, we present the complete genome sequence of a *bla*_OXA-58_-producing *Acinetobacter baumannii* strain, sampled from a Mexican hospital and not related to the international clones.

## GENOME ANNOUNCEMENT

*Acinetobacter baumannii* is a pathogen responsible for numerous infections and outbreaks in the clinical environment, and it is a major cause of morbidity and mortality in Latin America and in the rest of the world (1, 2). The abilities of this organism to acquire antibiotic resistance genes, to form biofilms, and to resist desiccation facilitate its permanence in the hospital setting and promote the emergence of outbreaks. Most of the nosocomial outbreaks worldwide are produced by a limited group of strains belonging to the international clones I and II (3).

In the last decade, this organism has steadily increased its resistance to carbapenems, an alarming situation because those antibiotics are one of the last-resort drugs for treating infections. Resistance to carbapenems in *A. baumannii* has been linked to the production of six groups of carbapenem-hydrolyzing class D β-lactamases: OXA-51-like, OXA-23-like, OXA-40/24-like, OXA-58-like, OXA-143-like, and OXA-235-like. Genes encoding OXA-51-like carbapenemases are encoded in the chromosome of almost all *A. baumannii* strains. The other five groups of carbapenemases are usually encoded within mobile genetic elements such as plasmids and transposons (4, 5).

*A. baumannii* strain 7804 was recovered in July 2006 from a bronchoalveolar lavage fluid specimen from a 25-year-old male patient admitted to the Hospital Universitario de Nuevo León, a tertiary care center (Nuevo León State, Mexico). A previous study showed that this strain is not susceptible to carbapenems and is not related to the international clones; it belongs to sequence type 490 (ST490) (Oxford scheme) and to clonal complex 110 (CC110) (6). The same study also indicated that this strain has an OXA-58-like gene associated with an ISAba3 element (6).

The genome sequence of this strain was determined with two single-molecule real-time (SMRT) cells on a PacBio RSII platform. Subreads were assembled *de novo* using the RS hierarchical genome assembly process (HGAP) protocol version 3, in SMRT analysis version 2.3 (Pacific Biosciences). The assembly has 98× coverage. Unitigs corresponding to the chromosome and plasmids were circularized using a Perl script (available at https://github.com/jfass/apc). Functional annotation was done with the NCBI Prokaryotic Genome Annotation Pipeline. The genome of *A. baumannii* 7804 contains one circular chromosome (4,159,217 bp) and two plasmids, pAba7804a (12,381 bp) and pAba7804b (170,420 bp). The chromosome has 6 rRNA operons, 75 tRNAs, and 3,892 coding sequences (CDS). The acquired antibiotic resistance genes were identified via ResFinder 2.1 (7) with the following results: the chromosome embraces one gene encoding aminoglycoside resistance *aph*(*3′*)-*Via* and two genes encoding β-lactamases genes (*bla*_ADC-25_ and *bla*_OXA-64_). Plasmid pAba7804b carries two genes related to aminoglycosid resistance (*strA* and *strB*), one sulfonamide resistance gene (*sul2*), and another gene related to tetracycline resistance (*tetB*). The *bla*_OXA-58_ gene is carried in the small plasmid. Two recently published genomes (8, 9) along with this strain represent the first *A. baumannii* genome sequences from Mexico and should be instrumental in the characterization of the genomic diversity of this nosocomial pathogen in this country.

### Accession number(s).

The genome sequence of isolate 7804 was deposited in GenBank under the accession numbers CP022283, CP022284, and CP022285.
